# Immunogenomics for identification of disease resistance genes in pigs: a review focusing on Gram-negative *bacilli*

**DOI:** 10.1186/2049-1891-3-34

**Published:** 2012-11-08

**Authors:** Shuhong Zhao, Mengjin Zhu, Hongbo Chen

**Affiliations:** 1 Key Laboratory of Agricultural Animal Genetics, Breeding and Reproduction of Ministry of Education, Huazhong Agricultural University, Wuhan, 430070, People’s Republic of China; 2 Hubei Key Laboratory of Animal Nutrition and Feed Science, Wuhan Polytechnic University, Wuhan, 430023, People’s Republic of China

**Keywords:** Pig, Gram-negative bacteria, Immunogenomics, Disease resistance breeding

## Abstract

Over the past years, infectious disease has caused enormous economic loss in pig industry. Among the pathogens, gram negative bacteria not only cause inflammation, but also cause different diseases and make the pigs more susceptible to virus infection. Vaccination, medication and elimination of sick pigs are major strategies of controlling disease. Genetic methods, such as selection of disease resistance in the pig, have not been widely used. Recently, the completion of the porcine whole genome sequencing has provided powerful tools to identify the genome regions that harboring genes controlling disease or immunity. Immunogenomics, which combines DNA variations, transcriptome, immune response, and QTL mapping data to illustrate the interactions between pathogen and host immune system, will be an effective genomics tool for identification of disease resistance genes in pigs. These genes will be potential targets for disease resistance in breeding programs. This paper reviewed the progress of disease resistance study in the pig focusing on Gram-negative bacilli. Major porcine Gram-negative bacilli and diseases, suggested candidate genes/pathways against porcine Gram-negative bacilli, and distributions of QTLs for immune capacity on pig chromosomes were summarized. Some tools for immunogenomics research were described. We conclude that integration of sequencing, whole genome associations, functional genomics studies, and immune response information is necessary to illustrate molecular mechanisms and key genes in disease resistance.

## Introduction

Infectious disease, caused by bacteria or virus, has always been a big barrier of effective pig production worldwide. The economics loss caused by infectious disease estimated to be RMB 40 billion annually in China. Gram negative bacteria, such as *Escherichia coli, Salmonella, Haemophilus parasuis* etc., has been a significant problem in pig industry. Pathogens generated by these bacteria not only cause inflammation, but also cause different disease and make the pigs more susceptible to virus infection. Different strains of *E. Coli* can cause piglet diarrhoea and enterotoxemia, which result in decreased weight gain or even sudden death [1]. *Salmonella enterica* can cause both pneumonia and diarrhea in the pig, as well as human food-borne gastroenteritis [2,3]. Another important harmful bacterium, *Haemophilus parasuis* (HPS), is a pathogen that can causes fibrinous polyserositis, meningitis and arthritis, called Glässer's disease [4].

Traditionally, producers use vaccination, medication and elimination of sick pigs as major strategies of controlling disease [1,2]. Genetic methods such as selection of disease resistance in the pig has not been widely used due to several reasons: First, selection of meat production traits ignored the improvement of disease resistance traits; Second, due to the complexity of infectious disease caused by multiple pathogens, selection hardly become effective to control all type of diseases; Third, basic research has not identified enough genes/pathways that can be used in disease resistant breeding.

Recently, the completion of the porcine whole genome sequencing [5], has provided powerful tools to dissect the genome that harboring genes controlling immunity. Over the past few years, large amount of data was generated through QTL mapping, expression profiling by microarray or expressed sequence tags, RNA-sequencing, and SNP chips in the pig. Immunogenomics, which combines DNA variations, transcriptome, immune response data, and QTL mapping to illustrate the interactions between pathogen and host immune system, will be an effective genomics tool for identification of disease resistance genes in pigs. These genes will be potential targets for disease resistance in breeding programs. This paper reviewed the progress of disease resistance study in the pig focusing on Gram-negative bacilli.

## General consideration of disease resistance

### Genetics of disease resistance

Understanding the genetics of disease resistance is a key issue in improving animal health through traditional or molecular breeding approach. Early studies demonstrated that resistance to infection of certain pathogen is heritable. Lundeheim [6] showed that the heritability for disease resistance to Enzootic pneumonia, Pleuritis, and Atrophic Rhinitis in the pig were 0.12, 0.13 and 0.16, respectively [6]. Occurrence of Enteric, respiratory, and chronic Pleuritis diseases was significantly different among Landrace, Yorkshire, Hampshire, and Duroc breeds [7]. Henryon et al. [8] found that heritabilities of respiratory diseases and diarrhoea are 0.24 and 0.30. These studies revealed additive genetic variation of resistance to certain pathogen does exist in different pig breeds, suggesting that selection in breeding for disease resistance could be efficient. However, there are many factors affect heritability estimation, e.g. different genetic backgrounds, environmental conditions can cause inaccuracy of heritability estimation, which could induce errors in breeding value estimation in using these parameters. Bishop et al. [9] showed that factors including incomplete exposure to infection, or imperfect diagnostic test sensitivity and specificity may reduce the estimable heritabilities, which reduces the power of datasets, but are not critical for illustration of host genetic differences in resistance.

Direct selection based on recovery after infection is not a practical way of disease resistance breeding. Moreover, focusing on one bacteria or virus cannot improve the disease resistance to multiple pathogens. Indicator traits, such as lymphocyte counts and proportions of various leucocyte subsets, innate immune response parameters, and adaptive immune response parameters, are high rank traits to improve the health status and disease resistance ability of pig populations. Thus, understanding the genetic basis of general resistance to multiple infectious diseases, and identification of indicator traits that can be used in breeding program are very important in future pig industry. Edfors-Lilja et al. [10] showed that heritability estimates of responses to *E. coli* antigens were ranging from 0.29 to 0.45. Mallard et al. [11] showed that heritability estimates in a Yorkshire population were 0.25, 0.23, 0.08 and zero for secondary antibody response to Hen Egg White Lysozyme, blastogenic response to Con A, serum IgG, and monocyte function(uptake and killing of *S. typhimurium*), respectively. Clapperton et al. [12] demonstrated that significant differences existed between Meishan and Large White pigs for a number of innate immune traits, e.g. total white cell counts were similar between the pig breeds but the numbers of lymphocytes, neutrophils and monocytes differed significantly in that Meishans having higher neutrophil and monocyte counts and lower lymphocyte counts. Juul-Madsen et al. [13] showed that serum porcine mannan-binding lectin A (pMBL-A) concentration is significantly higher in Landrace breed than that in Duroc breed, and the heritability of pMBL-A level is high in the Landrace (*h*^*2*^=0.8) but not in the Duroc breed (*h*^*2*^=0.15). pMBL-A is an innate immune collectin binding to microbial carbohydrates, higher concentration enhance pathogen killing and clearance. Flori et al. [14] scored and analyzed heritabilities of a number of immunity parameters three weeks after vaccination against Mycoplasma hyopneumoniae in a French Large White population, the results showed that 42 of the 54 measured parameters showed moderate to high heritabilities (≥0.2). However, relationship between improved levels of these parameters and disease resistance has not been illustrated clearly, e.g. how to define a pig is resistant, yet deciding of the levels of the innate immune factors that can keep the pigs healthier is of great challenging.

Genetic parameter estimation and application based on phenotypic records remains a big challenge in selection for disease resistance in the pig. Moderate to high heritability of some traits indicates there are genes/markers controlling these traits in the genome remain identified. Genomics tools, including genetic and physical maps, sequencing and expression studies offered powerful tools to discover genetic markers and genes that can be used in improvement of disease resistance. More genes have been identified in mice comparing to farm animals due to the advance of genomic tools and application of inbred strains. In the pig, few genes have been identified to control disease. A typical example is the presence or absence of the receptor of K88, a cell-surface antigen on some Escherichia coli, can cause diarrhoea in the pig [15,16]. Alleles in mucin genes have been shown to have strong association with susceptibility to enterotoxigenic Escherichia coli F4ab/ac in the pig [17,18]. Unfortunately, not many pathogens have an inheritance pattern as the *Escherichia coli* F4, there are more genes and complicated pathways involved in controlling their infections. It is urgent to use immunogenomics tools to uncover the useful genes and pathways to specific pathogen resistance and enhance the innate immunity in different pig breeds.

### Strategies for revealing genes and molecular mechanisms of disease resistance

There are different ways to identify genes and molecular mechanisms in disease resistance. However, no single approach can effectively identify genes and the controlling pathways. Integration of sequencing, whole genome associations, functional genomics studies, and immune response information is necessary to illustrate molecular mechanisms and key genes in disease resistance.

#### Candidate gene approach

In a previous review, we have described the progress and challenges of candidate gene approach [19]. As mentioned above, many diseases have genetic basis, identifying the major genes that play roles in controlling these diseases is of great interest to researchers. Candidate genes are genes with known biological function that directly or indirectly regulating the developing processes of the investigated disease. With the modern developed sequencing techniques today, these studies are relatively cheap and quick to perform. Moreover, the selected genes have been shown to be related to disease on the biology function from previous studies, thus it is more likely to find associations with the target disease traits. Typically, case/control studies from susceptible or resistant animals, or different livestock breeds were carried out to identify mutations in the candidate genes. The basic idea is to analyze the mutations in susceptible / resistant animals, or different breeds with different susceptibility to a certain infection. Alleles based on the mutations find in these genes may be useful markers for disease resistance breeding. In the pig, a number of studies have been focused on immune response and disease resistance traits (See 3.3).

#### Genome wide scan approach

Many traits, including disease resistance and immune response traits, are quantitative traits that usually determined by multiple genes. Candidate gene approach is largely limited in identifying multiple genes simultaneously that contribute to a phenotype. Thus, genome wide scans to identify chromosome regions and genes have been applied widely. Many QTL studies have been conducted in the pig [20-23]. A number of QTLs associated with a single trait were identified. (http://www.animalgenome.org/cgi-bin/QTLdb/SS/summary? summ=type&qtl=6433&pub=283&trait=594). A total of 40 QTLs were identified for *Samonella* count in liver and spleen [24]. Six QTLs were identified for resistance to *E. coli*[25]. No studies were attempted to identify QTLs for other gram negative bacteria. This may be due to the difficulty of creation of QTL mapping populations. QTL studies offered good clue for further cloning of the genes that controlling a trait. However, for the identified QTLs in livestock, a large number of them were located in relatively large chromosome regions,which brings difficulty of positional cloning of the key genes or QTNs for a certain trait. Also, the rather low accuracy of QTL locations is a big challenge of using them in breeding programs. Further, the interactions of the QTLs remain largely unclear, which leads to uncertain results when using them in selection.

Along with the completion swine genome sequencing and development of the high throughput SNP chip, genome wide association study (GWAS) tools became available in identification of key genes associated with disease resistance traits. The analytical tools of GWAS provided us powerful means to identify the possible variants underlying the target traits at the whole-genome scale. In the past years, the applications of GWAS in identification of susceptibility/resistance loci for a range of diseases and resistance-related traits have achieved great progress. Despite of obvious merits, GWAS currently has some limitations in its applications for pig disease studies, which includes high costs, common hypothesis-dependence and other ones that traditional candidate gene studies meet. Especially, one of the disadvantages of GWAS is that the association results are rarely supported by studies on the identification of causal variants or on the functional characterizations of putative SNPs. In addition, with development of genome techniques and accumulation of porcine genome resources, systems biology approach provides a new strategy for identification of disease resistance loci in pigs. In the near future, it is anticipated that systems biological approaches would encourage and accelerate the steps of pig disease resistance investigations.

## Genetic control of porcine resistance to Gram-negative bacilli infections

### A brief introduction of porcine Gram-negative bacilli

Although it is somewhat ambiguous, we normally describe bacteria as Gram-positive and Gram-negative. Gram-negative bacteria do not retain crystal violet dye after adding a counterstain, such as safranin, in the Gram staining protocol, thus are colored with a red or pink color. Lipopolysaccharide (LPS), also known as endotoxin, is an essential constituent of the outer Gram-negative cell wall. Lipid components such as Lipid A and polysaccharide are closely associated with the toxicity and antigenicity of Gram-negative bacteria, respectively [26]. Pathogenic Gram-negative bacteria, e.g. *Salmonella*, *Escherichia coli*, have Pathogenicity Islands (PAI) on the bacterial chromosome, which play a pivotal role in the virulence [27]. PAIs are found mainly in Gram-negatives, but have been reported in a few Gram-positive bacteria. For more details, readers can reference Schmidt and Hensel (2004) [28]. Based on cell shape, Gram-negative bacteria comprise three major subdivisions: coccobacilli, cocci, and baciili. In pigs, there are many Gram-negative bacilli of clinical significance (Table 1) besides the species mentioned above. These include species of the family Enterobacteriaceae (e.g. *Salmonella enterica*, *Escherichia coli*, *Klebsiella pneumoniae*, and *Yersinia* species). Infection with these members may or may not lead to high mortality, but affects pig industry world-wild and leads to significant economic losses. Additionally, they are causative agent (especially the salmonella and *Escherichia coli*) of food borne illness in humans, which has become a severe public health problem. Another bacillus of public health significance is *Campylobacter coli*, which belongs to the family of Campylobacteraceae and causes enteritis in pigs. Infection with *Lawsonia intracellularis* causes proliferative hemorrhagic enteropathy (PHE), which has become one of the most economically important diseases in pigs [29]. Production of PHE result in bloody diarrhea and sudden death in adult pigs; but in growing pigs, *Lawsonia intracellularis* infection causes chronic proliferative enteropathy (PE) (reviewed in [30]). Other important Gram-negative bacilli include *Actinobacillus pleuropneumoniae* and *Haemophilus parasuis*, two bacilli of the family of Pasteurellaceae. *Actinobacillus pleuropneumoniae* is the causative agent of porcine pleuropneumonia and fibrinous pleuritis [31,32]. *Haemophilus parasuis* infection also leads to respiratory diseases such as acute pneumonia, but the major clinical manifestation is Glässer's disease [33]. In brief, Gram-negative bacilli that have great impacts on pig industry are mainly classified into two categories: 1) the enteric agents that are closely associate with diarrhea; 2) the members of Pasteurellaceae family that cause pneumonia and other severe inflammations.

**Table 1:** Major porcine Gram-negative bacilli and diseases

**Causal bacterium**	**Family; Genus**	**Pig diseases**	**Reference**
*Salmonella enterica* (*S. enterica*) (serovar *Choleraesuis* and *Typhimurium*)	Enterobacteriaceae; *Salmonella*	common diseases with diarrhea and enterocolitis	[34,35]
*Escherichia coli* (*E. coli*)	Enterobacteriaceae; *Escherichia*	diarrhea, hemorrhagic colitis (HC), and hemolytic-uremic syndrome (HUS)	[36,37]
*Klebsiella pneumoniae* (*K. pneumoniae*)	Enterobacteriaceae; *Klebsiella*	diarrhea, septicaemia, sudden death especially in preweaned pigs	[38,39]
*Yersinia* species (*Y. pseudotuberculosis*; *Y. enterocolitica*)	Enterobacteriaceae; *Yersinia*	enteritis (or diarrhea)	[40-42]
*Actinobacillus pleuropneumoniae* (*A. pleuropneumoniae*)	Pasteurellaceae; *Actinobacillus*	fibrinohemorrhagic necrotizing bronchopneumonia, fibrinous pleuritis, acute pleuropneumonia mainly in growing pigs	[31,32]
*Haemophilus parasuis* (*H. parasuis*)	Pasteurellaceae; *Haemophilus*	Glässer's disease, acute pneumoniae and acute septicaemia	[33]
*Lawsonia intracellularis* (*L. intracellularis*)	Desulfovibrionaceae; *Lawsonia*	chronic diarrhea in young growing pigs, proliferative hemorrhagic enteropathy (PHE)	[43,44]
*Campylobacter coli* (*C. coli*)	Campylobacteraceae*; Campylobacter*	enteritis	[45]

### Resistance indicator for Gram-negative bacilli

Complete resistance to Gram-negative bacilli would be expected in pigs that have capability of 100% elimination, similar to the resistance to other pathogenic invaders e.g. virus, parasites, and Gram-positives. In order to fight infection, vertebrates including the pig own three lines of defense. The first line of defense such as the skin and mucosae provides physical and chemical barriers. In most instances, these barriers unfortunately do not work very well. Severe respiratory diseases and intestine problems caused by Gram-negative bacilli (Table 1) are obvious examples for this. The failed guarding of the first line results in colonization and deeper host tissue invasion, which is mediated by several complex mechanisms [46].

In fact, resistance has happened once host-pathogen interactions begin. Gram-negative bacilli-resistant pigs have strong capability of elimination, due mainly to the effectiveness of the host immune response (IR) mechanisms. The earliest resistance indicators are developed during innate immunity, which is the second line of defense. Gram-negative cell wall components (LPS, porins in the outer membrane, and peptidoglycan monomers etc.), which are collectively called pathogen-associated molecular patterns (PAMPs), play critical roles in host initial IRs [47]. Generally, bindings of PAMPs to pattern-recognition receptors (PRRs) on a variety of host defense cells (e.g. macrophages, neutrophils, and epithelia) result in secretions of many cytokines (e.g. tumor necrosis factor-alpha, interleukin-1, and interleukin-8), which in turn lead to protective inflammatory processes, phagocytosis, and activation of the complement classical pathways and the coagulation pathway [47]. Uthe et al. [48] showed that innate immunity and the inflammatory T helper 1 (Th1) response were observed during both *Salmonella enterica* serovar *Choleraesuis* infection and serovar *Typhimurium* infection. Our previous results suggested that *Haemophilus parasuis* infection also engaged immune-inflammatory mechanisms [4,49]. These early resistance indicators belong to nonspecific immune responses but are critical for detecting non-selves and clearing infection. On the other hand, some bacilli infections such as *E. coli* and *Y. pseudotuberculosis* can inhibit complement, attenuate host inflammatory response [50], and reduce the recruitment of professional phagocytes [51], indicating these host resistance indicators are main targets in immune evasion mechanisms of Gram-negative bacilli. To better fight against invaders, pigs have to use the last line of defense which is called adaptive immunity. Antibodies produced by plasmacytes are major mediators of adaptive immunity; they are involved in a wild range of host protective IRs including opsonophagocytosis, MAC cytolysis, neutralization, and ADCC (antibody-dependent cell mediated cytotoxicity), so it is the most effective defense by host. For example, this has been proved by the fact that maternal antibodies play important roles in protection against *H. parasuis* infection [52,53]. Protective antibody responses has also been observed during *A. pleuropneumoniae* infection [54,55]. Guedes and Gebhart [56] showed that cell-mediated immune response was weak during *L. intracellularis* infection but specific local intestinal humoral IR mediated by IgG was observed.

### Candidate gene identification by genetics and omics approaches

A critical goal of porcine genome research is the development of genomic-based tools to select for disease resistance/susceptibility and improved health traits. Although much consideration is needed, good news is that variations in resistance to many porcine pathogens including Gram-negatives do exist [57-60]. For breeding programs such as MAS, the first task is to attempt to identify host candidate genes/genetic markers. With the development of genomics, both traditional methods (e.g. QTL mapping) and state of the art approaches (e.g. GWAS) have been pursued to understand genetic control of host resistance to various causing agents. To date, majority studies have been focused on Gram-negative bacilli infections such as *E. coli*, *S. enterica*, *A. pleuropneumoniae*, and *H. parasuis*, whereas few have been done on other kind of bacilli (Table 2). This may due to the importance of some bacilli in pig industry.

**Table 2:** **Suggested candidate genes/pathways against porcine Gram-negative bacilli**^**§**^

**Causal bacterium**	**Tissue/Organ**	**Suggested candidate gene/pathway or major conclusion**
*S. Typhimurium*	*	*HP*, *NCF2*, *PGD*[3]
	intestine (jejunum, ileum and colon)	*TLR-2*, *NOD-1*, *NOD-2*, *PBD-2*, *NF-κB1*, *caspase-1* Regional differences in gene expression profiles and inflammatory response to *S. typhimurium* infection along the porcine intestinal gut exist [64]
	macrophage	Enhanced uptake of *S. typhimurium* in macrophages is associated with ERK1/2 activation [63]
	intestinal epithelial cell	*PBD-1*, *PBD-2*[61,67,68]
	*	*CCT7*[69]
	in vivo gut loop model	*NOD-2*, *TLR-2*, *TLR-4*, *TLR5*, *CCR9*, *CCRL1*[65]
	mesenteric lymph node	T helper 1, innate/inflammatory, and antigen-processing pathways are induced; apoptosis and antigen presentation/dendritic cell function pathways are down-regulated; NF-kappaB suppression in antigen-presenting cells may be the mechanism for *S. Typhimurium* evasion [62]
	mesenteric lymph node	*CD47*, *CXCL10*, *SCARB2*, *INDO*, *IRF1*, *SOCS1*, *STAT1*, *SLC11A1*[47]
*S. Choleraesuis*	*	14 different chromosomal regions in the porcine genome are found to be significantly associated with susceptibility [24]
	mesenteric lymph node	Th1, innate immune/inflammation response, apoptosis pathway, and strong NF-kappaB-dependent response are induced [35]
	mesenteric lymph node	*ARPC2*, *CCT7*, *HSPH1*, *LCP1*, *PTMA*, *SDCBP*, *VCP*, *INDO*, *SOCS1*, *STAT1*, *SLC11A1*[48]
	lung	*TGM1*, *TGM3*, *GBP1*, *GBP2*, *C1S*, *C1R*, *MHC2TA*, *PSMB8*, *TAP1*, *TAP2*
		Apoptotic pathways, Th1 immune response, and interferon gamma (IFNG) signal are observed [2,66]
*E. coli*	*	*HEG1*, *ITGB5*[70]; *MUC4*[71,72]; *FUT1*[73,74]; *B3GNT5*[75,76]; *MUC20*[77]; *MUC13*[18]; *TFRC*[75,78]; *B3GALT3*, *B4GALT4*[75]
	duodenum	Genes related to the Glycan Biosynthesis and Metabolism are observed [79]
	Jejunal mucosa	THO complex 4 [80]
*A. pleuropneumoniae*	lung	*MMP-9*, *MMP-12*[81]
	liver	liver plays an important role in initiating and orchestrating the innate immune response to *A. pleuropneumoniae* infection [82]
	*	*TF*[83]
	peripheral blood leukocytes	*OAS1*, *CD97*, *S100A8*, *TGM3*[84]
	lung, liver, tracheobronchial lymph node	357, 713, and 130 differentially expressed genes are observed in lung, liver, and tracheobronchial lymph node, respectively (For more details see [85])
*H. parasuis*	porcine alveolar macrophage	*S100A4*, *S100A6*, coronin1a, etc. Cell adhesion molecules, cytokine-cytokine receptor interaction, complement and coagulation cascades, toll-like receptor signaling pathway, and MAPK signaling pathway are significantly effected [86]
	*	*FUT1 *[87]
	lung	Candidate genes and pathways for disease resistance or susceptibility phenotypes are identified (For more details see [88])
	*	*CAV1*[89]
	spleen	*S100A8*, *S100A9*, *RETN*, etc. [4,49]
*L. intracellularis*	intestinal tissue	*IGFBP-3*[90]
*K. pneumoniae*	N/A	N/A
*Y. pseudotuberculosis*	N/A	N/A
*Y. enterocolitica*	N/A	N/A
*C. coli*	N/A	N/A

§: all the data was obtained from PubMed (http://www.ncbi.nlm.nih.gov/pubmed).

* : Genetic analysis including bioinformatics SNP (Single Nucleotide Polymorphism ) prediction analysis, SNP and association analysis with traits, GWAS (Genome Wide Association Studies), and gene function analysis.

N/A: not available.

*S. Choleraesuis* and *S. Typhimurium* are the most commonly isolated serovars that effecting performances in pigs [61]. NF-kappaB pathway, antigen-presentation, ERK1/2 activation, and apoptosis involved in both innate and adaptive IRs are associated with infections [2,35,62,63]. Interestingly, *in vivo* and *in vitro* studies from different groups showed different or even contradictory results as indicated in expression patterns of PAMP receptors and porcine β-defensins (*PBD-1*, *PBD-2*) [64,65]. Additionally, IR patterns were different due to different subspecies infections and samplings (Table 2); differences in gene expressions exist even in different intestine regions [64]. Results above implied that host IRs are complex and co-works are usually necessary in resistance to *S. enterica* infections. This is consistent with the theory that most disease resistance traits are quantitative traits. Based on bioinformatic SNP predictions, Uthe et al. [3] revealed that SNPs in *HP*, *NCF2*, *PGD* were associated with Salmonella shedding. In our lab, Ma et al. [66] identified SNPs in GBP family genes that differentially expressed under *Salmonella* infection, and showed the SNPs were associated with blood parameter traits.

Thanks to candidate gene identification, associations between SNPs and *E. coli* F4ab/F4ac susceptibility have been extensively studied. Previous studies revealed that the loci controlling F4ab/F4ac receptor is located on SSC13q41 (reviewed in [70]). Mutations in functional genes including *HEG1*, *ITGB5*[65], *MUC4*[71,72], *FUT1*[73,74], *B3GNT5*[75,76], *MUC20*[77], and *MUC13*[18] were found to be associated with porcine susceptibility. Jacobsen et al. [91] characterized 34 SNPs in five candidate genes within the ETEC F4ab/ac candidate region, but no obvious causative mutations were identified for *E. coli* F4ab/F4ac susceptibility. These associations may be helpful for genetic improvement of porcine disease resistance to this bacterium.

Several groups have focused on candidate gene identifications following *A. pleuropneumoniae* and/or *H. parasuis* infections, but few mutations have not been revealed (Table 2). Daniłowicz et al. [83] identified 62 polymorphisms in *TF* genome sequence on a panel of 10 different pig breeds, but only found one possible association of the severity of *A. pleuropneumoniae* infection with TF genotypes. *Cav1* was differentially expressed under *H. parasuis* infection, SNPs in this gene were found associated with blood parameter traits [89].

**Figure 1:**
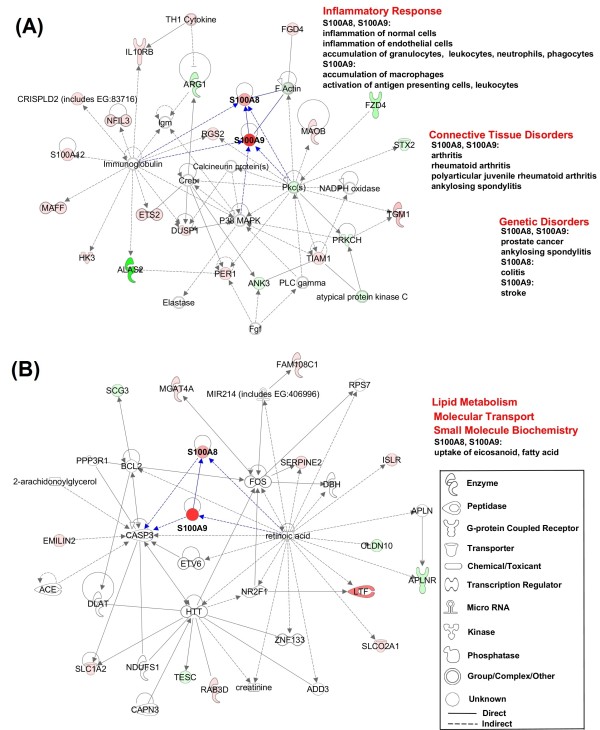
**Gene-interaction network analysis of porcine *S100A8 *and *S100A9 *in *H. parasuis *infected spleen.** Depicted are the results of the functional analysis of networks **A**, **B** in which porcine *S100A8* and *S100A9* are involved. The intensity of the node color indicates the degree of up- (red) or down- (green) regulation. Nodes are displayed using various shapes that represent the functional class of the gene product as indicated in the list in B [49] .

As for candidate gene identification, much consideration should be given in the future. First, identification of candidate genes needs to be strengthened by employing high throughput approaches, bioinformatics, and functional genomics. In response to pathogenic agents, different kinds of host IRs would be induced or inhibited simultaneously. It is a challenge to attempt to interpret tens of thousands data and then identify the right genes against diseases. Our previous work on *H. parasuis* infection revealed systematic changes of gene pathways/networks during the process. In combining with gene function analysis, one selective way is to try to identify the main actors that closely related to clinical signs (Figure 1), but this may not easy to do especially when lacking of powerful tools. Some approaches such as GWAS can help us to identify genetic markers directly but the following analysis on gene functions (if it is not available) is usually necessary. This is not only let us to know “what” but also “how” the candidate genes play roles in porcine resistance/susceptibility. Second, candidate genes resistant to multiple pathogens are more useful. In most cases, multiple pathogens play roles simultaneously in porcine infectious diseases. For example, *Actinobacillus pleuropneumoniae*, *Pasteurella multocida* and *Staphylococcus aureus* in porcine lung infections [81]; and, presence of pathogenic porcine reproductive and respiratory syndrome virus (PRRSV) accelerates *H. parasuis* infection [92]. Third, animal infection models are mainly but not limited in pigs. Since some Gram-negative bacilli have many hosts including human and mouse, host functional genes in other species might also be good references for pig research. An example for this is that Oh et al. [93] has revealed cell cycle and cell differentiation genes in mouse intestinal crypt epithelial cells following *L. intracellularis* infection. In brief, the candidate gene approach has been widely used to identify genes, but very limited mutations have been found to be powerful in breeding schemes. A number of mutations need to be tested in large field data set. Moreover, only a few genes and a small number of mutations have been preliminarily studied. High throughput strategies (e.g. SNPchip and deep sequencing) and bioinformatic meta-analysis enable researchers to identify genes in a more efficient way.

## Database resources for livestock immunomic research

One of the characteristics of contemporary life science is that most of the research results have been digitalized and deposited in public or specific databases, and are able to be traced. Up to date, there are a series of different types of digital repositories for disease resistance or immunity-related resources, which vary from phenotype to DNA, to mRNA, or to protein. These resources can be easily retrieved and widely applied to accelerate our own researches on livestock immunogenomics.

### AnimalQTL database

**Figure 2:**
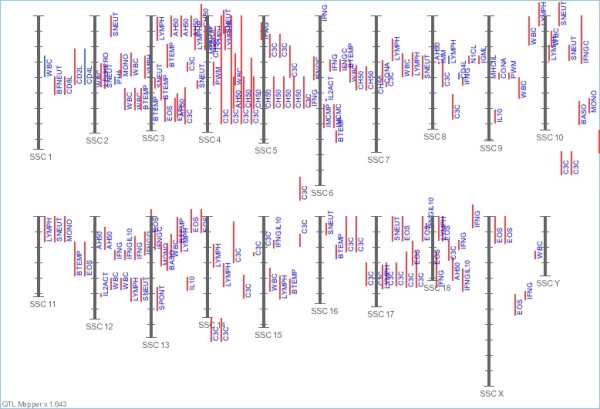
**Distributions of QTLs for immune capacity on pig chromosomes.** The red lines represent for significant QTLs and the blue ones for suggestive statistical evidence (This figure is generated from AnimalGenome.ORG with kind permission).

The AnimalQTL database, led by Iowa State University under the NAGRP Bioinformatics Coordination Program, has gathered the published QTLs identification results from the species of pig, cattle, chicken, sheep and rainbow trout. There are totally 640 health-related QTLs deposited in the division of PigQTLdb, in which containing 174 QTLs for immune capacity (e.g., CD4-positive leukocyte number, interferon-gamma level, eosinophil number, monocyte number, and lymphocyte percentage), 107 for disease susceptibility (e.g., chronic pleuritis, melanoma susceptibility, pseudorabies susceptibility, and PRRSV antibody titer), 43 for pathogen (e.g., parasite load and Salmonella counts), and some for blood parameters (e.g., mean corpuscular volume, hematocrit, haptoglobin concentration, and red cell distribution width). It can be found that the QTLs of health traits have located across all porcine chromosomes, some of which are locally clustered. Figure 2 displayed the distributions of QTLs for immune capacity on pig chromosomes. The database resources of disease or immunity-related QTLs provide us useful reference information when mining of resistance alleles.

### KEGG database

KEGG (Kyoto Encyclopedia of Genes and Genomes) is a widely-used digital resource that contains the regulatory relationship between molecules/genes based on the network/pathway forms (KEGG pathway maps, BRITE functional hierarchies, and KEGG modules). KEGG provides several entries to extract the disease or immunity-related resources. In KEGG DISEASE, the molecular networks of different types of diseases, including single-gene (monogenic) diseases, multifactorial diseases such as cancers, immune system diseases, neurodegenerative diseases, cardiovascular diseases, metabolic diseases, and infectious diseases, can be searched. The KEGG PATHWAYDatabase provides a collection of manually drawn pathway maps according to the functional classifications such as metabolism, genetic information processing, cellular processes, organismal systems, and diseases. Immune system under the organismal systems contains a series of immune signaling pathways, in which including Hematopoietic cell lineage, Complement and coagulation cascades, Toll-like receptor signaling pathway, NOD-like receptor signaling pathway, RIG-I-like receptor signaling pathway, Cytosolic DNA-sensing pathway, Natural killer cell mediated cytotoxicity, Antigen processing and presentation, T cell receptor signaling pathway, B cell receptor signaling pathway, Fc epsilon RI signaling pathway, Fc gamma R-mediated phagocytosis, Leukocyte transendothelial migration, Intestinal immune network for IgA production, and Chemokine signaling pathway. Most of the immune signaling pathways are mainly organized by species (including human, chimpanzee, rat, pig, dog, chicken, mouse, cow, zebrafish, and so on). KEGG provides a reference knowledge base for integration and interpretation of large-scale data produced from genome sequencing and other high-throughput experiments [94], which can be also applied to provide the molecular network-based views of diseases resistance.

### Interactome databases

**Figure 3:**
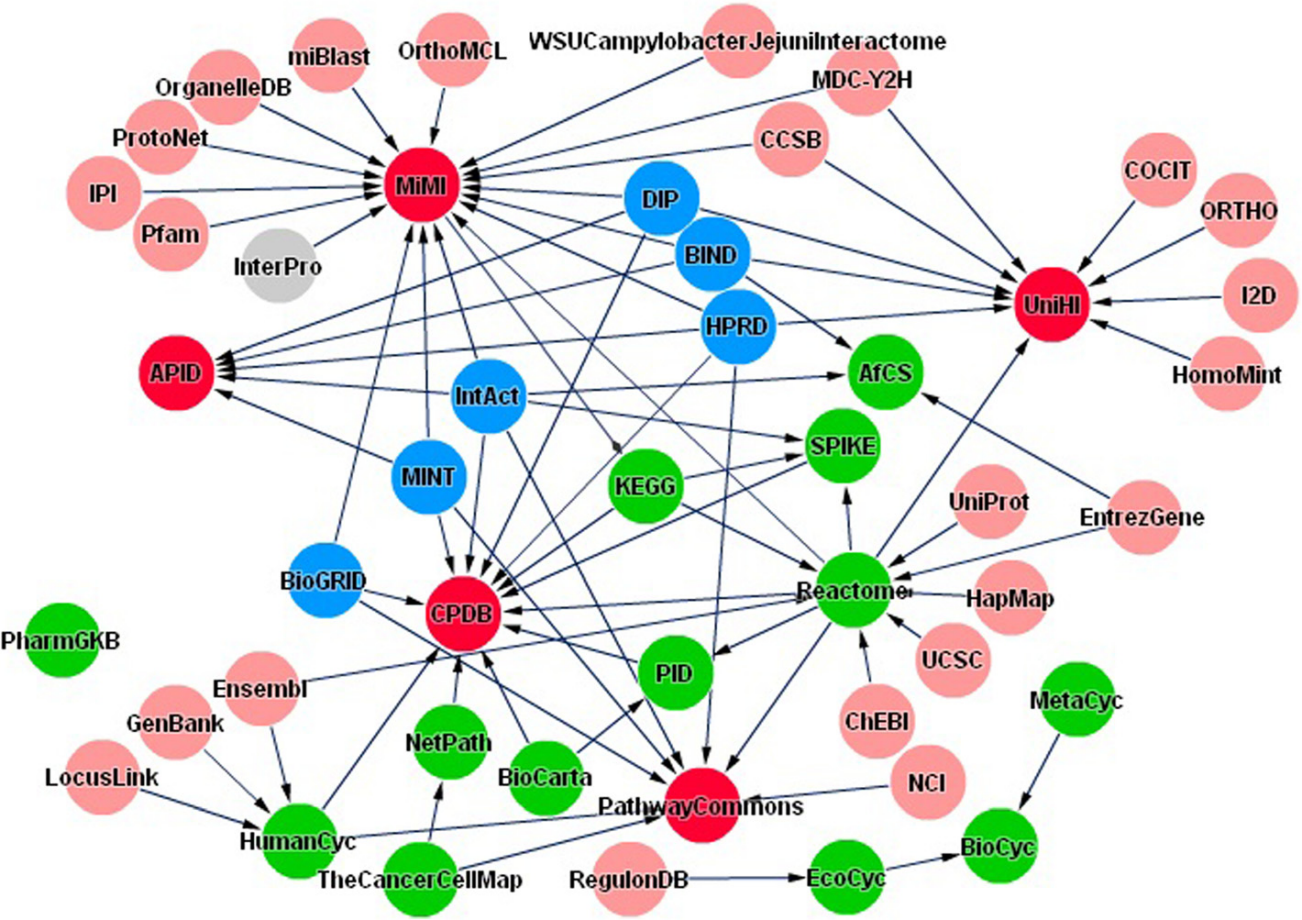
**The links among databases in Pathguide.** (This figure is generated from the Pathguide website).

There are many interactome databases such as APID, BioGRID database, Bioverse database, ConsensusPathDB, MIPS database, PSIMAP database, InterPare database,Biomolecular Interaction Network Database (BIND), Online Predicted Human Interaction Database (OPHID), Human Protein Reference Database (HPRD), Human Protein Interaction Database (HPID), Molecular INTeraction database (MINT), Proteins Interacting in the Nucleus Database (PINdb), Molecular Interaction Database (IntAct), VirHostNet, Interactome Databases at CCSB,TRANSFAC, and PSIbase Database. Most of these interactome databases are not independent, the website of Pathguide has displayed the relationships between some databases (Figure 3). These databases contain a large volume of molecules involving disease or immunity, especially the interactions between protein molecules, which also provide references for interpretating and understanding the molecular bases of disease resistance.

### Microarray databases

Functional genomics is one important aspect of livestock immunogenomics. Microarray experimental techniques have largely and historically, and now still, contributed to the studies on livestock immunogenomics, which produced a large amount of microarray resources on Internet, also including exons arrays and RNA-seq data. There are many microarray databases including the most known public database, the Gene Expression Omnibus (GEO) from NCBI or ArrayExpress from EBI. In addition, some specific or curated databases also host microarray data repositories for disease or immunity such as the Immunological Genome Project, caArray at NCI, ImmGen database, and Stanford Microarray Database. Obviously, functional information or molecular bases of disease resistances can be mined or re-mined from these microarray databases.

## Prospects on livestock disease resistance research

Different from other economic traits, it is difficult to make direct measurements of the traits or indicators of disease resistances that are ordinarily not measurable or unknowable on most occasions. Exactly speaking, most of the disease resistances, usually belonging to threshold or quantitative traits under polygenic, genetically heterogeneous control with a small proportion of additive genetic effects, are essentially revealed by death rate or mortality rate when exposed to disease pathogens. But, in practice, it is high cost and difficult to operate the direct measurement of disease resistance and thus disease resistances are alternatively measured by immunity-related traits. Thus, the immune traits are closely associated but not directly with the measurements of disease resistance. Nevertheless, there are so many immune traits, and it is still unknown which immune trait is the best one for measuring the disease resistance. There is an urgent need to find an immune trait or a restructured indicator to pinpoint or be possible mostly near the disease resistance. Currently, although microarray techniques have been widely used to investigate the molecular basis of disease resistance, other genomic approaches such as proteomics and metabonomics/metabolomics are rarely involved in. Furthermore, because of the complexity, high cost, lack of available data, and diversity of host-pathogen interaction, computational aspects of disease genomics are still in challenge. In this field, new and innovative research approaches such as novel genomic and systems biological techniques will be heavily dependent and applied more widely in future. In our opinion, one important aspect of the future studies should focus on Chinese indigenous pig breeds that deposit a large complex of gene resources for disease resistance, including isolation, cloning, and identification of specific resistance alleles accompanying with large-scale explorations of their biological functions. Besides mining gene materials for anti-disease breeding, we should also eye on the application-oriented issues such as molecular techniques for gene diagnosis and testing reagent boxes, and molecular selection approaches matching the characteristics of pig breeding system.

## Conclusions

Currently, immunotherapies, such as inoculation or immunization, and medicine administration are the main control strategies on pig diseases. The recently emerged strategy of disease resistance breeding is still immature, which is far from the actual applications. Compared with other economic traits, little breakthrough in disease resistance studies has been achieved. The limited progress and fragmentary results in this field cannot underpin an efficient application of genetic improvement programs to disease resistance. Aiding with novel genomic and systems biological techniques, such as high throughput sequencing, GWAS, and gene function analysis, will help to uncover the disease resistance genes and strengthen the studies of pig disease resistance. It is believed that disease resistance breeding will benefit the future pig industry.

## Competing interests

The authors declare that they have no competing interests.

## Authors’ contributions

SHZ completed the introduction and the first part of the paper, MJZ drafted the second part, HBC completed the third part of the paper. All authors read and approved the final manuscript.
